# Involvement of KEAP1/NRF2 pathway in non‐BRAF mutated squamous cell carcinoma of the thyroid

**DOI:** 10.1002/path.6444

**Published:** 2025-07-02

**Authors:** Elin Schoultz, Jakob Dahlberg, Lisa M Nilsson, Jozefina J Dzanan, Therese Carlsson, Niklas Dahr, Ellinor Andersson, Ghayeb Muhammad, Andreas Muth, Erik Elias, Henrik Fagman, Volkan I Sayin, Jonas A Nilsson, Mikael Nilsson

**Affiliations:** ^1^ Sahlgrenska Center for Cancer Research University of Gothenburg Göteborg Sweden; ^2^ Department of Medical Chemistry and Cell Biology, Institute of Biomedicine University of Gothenburg Göteborg Sweden; ^3^ Department of Surgery, Institute of Clinical Sciences University of Gothenburg Göteborg Sweden; ^4^ Region Västra Götaland, Department of Surgery Sahlgrenska University Hospital Göteborg Sweden; ^5^ Department of Clinical Genetics and Genomics Sahlgrenska University Hospital Göteborg Sweden; ^6^ Department of Clinical Pathology Sahlgrenska University Hospital Göteborg Sweden; ^7^ Department of Laboratory Medicine, Institute of Biomedicine University of Gothenburg Göteborg Sweden; ^8^ Harry Perkins Institute of Medical Research University of Western Australia Perth WA Australia

**Keywords:** PTC, ATC, SCC, squamous, PDX, KEAP1, NFE2L2, NRF2, NQO1, glutaminase inhibition

## Abstract

Squamous cell carcinoma (SCC) of the thyroid is a rare tumor that is classified as an anaplastic thyroid cancer (ATC) due to its similar unresponsiveness to chemoradiotherapy and an outstandingly poor prognosis. Due to its rarity, current knowledge about this tumor is mostly based on single‐case reports. The tumor‐cell‐origin and molecular pathogenesis remain unclear, although the presence of *BRAF* mutations in some cases suggest it may evolve from papillary thyroid carcinoma (PTC). Here we provide direct evidence of derivation of SCC of the thyroid from PTC, based on a unique combination of likely pathogenic mutations in *KEAP1*, *STK11* (*LKB1*), and *RB1* found in both tumor components, along with loss of one copy of chromosome 11 and additional somatic mutations in the SCC tumor. Transdifferentiation from PTC to SCC was also evident by immunohistochemistry. Out of eight attempted patient‐derived xenografts (PDX) from advanced thyroid cancers, only one derived from thyroid SCC successfully engrafted in immunodeficient NOG mice. Untreated PDXs showed high Ki67 indices but did not reproduce the conspicuous stromal invasion of CDH1^low^/SNAI2^+^/CDH2^+^ cells that characterized the primary tumor. Based on the mutation profile (*NFE2L2*, *PIK3CA*, *CDKN2A*, and *TP53*), experiments were designed to evaluate targeted drug therapy using third‐passage PDX transplants. The combination of TRK and PI3K inhibitors, cabozantinib and GDC‐0326, additively reduced PDX growth by nearly 90%. Remarkably, CB‐839 (telaglenastat), a glutaminase inhibitor targeting metabolic rewiring downstream of NRF2 activation, was equally effective. Both combined treatment with cabozantinib + GDC‐0326 and CB‐839 monotherapy diminished the expression of NQO1, an NRF2 transcriptional target, in tumor cells. Glutaminase inhibition further promoted squamous differentiation in engrafted tumors. Both investigated SCC tumors were negative for BRAFV600E or any other common driver mutation of thyroid cancer. Collectively, these findings indicate that aberrant activation of the KEAP1/NRF2 pathway due to somatic mutations is a previously unrecognized feature of thyroid SCC and suggest that glutaminase inhibition may serve as a potential therapeutic option for this subgroup of ATC patients. © 2025 The Author(s). *The Journal of Pathology* published by John Wiley & Sons Ltd on behalf of The Pathological Society of Great Britain and Ireland.

## Introduction

Squamous cell carcinoma (SCC) of the thyroid gland was recently reclassified to a subtype of anaplastic thyroid cancer (ATC) [1]. It is conceivable that thyroid SCCs either develop by transdifferentiation from a differentiated thyroid carcinoma (DTC) or arise *de novo* from a putative oligopotent ancestral mutant cell. However, with the exception of concurrent BRAFV600E mutation suggesting transition from a preexisting papillary thyroid carcinoma (PTC) [2, 3], the mutation status and tumor features of thyroid SCC are overall poorly documented [4]. The possibility of collision tumors is sometimes considered [5], and it is often challenging to exclude a nonthyroid tumor‐cell‐origin, such as nearby squamous epithelial organs, primarily the larynx, hypopharynx, or esophagus, from which an SCC tumor potentially might infiltrate by direct extension or metastasize to the thyroid.

Here we provide proof of concept that non‐BRAF mutated SCC of the thyroid may develop from preexisting PTC, involving homozygous inactivation of several tumor suppressor genes, including *KEAP1*, which has previously been reported to be oncogenic in lung adenocarcinoma [6] and lung SCC [7]. We also present experimental evidence of tumor growth inhibition in patient‐derived xenograft (PDX) models following drug therapy targeting metabolic vulnerabilities downstream of constitutive NRF2 activation in a thyroid SCC harboring *NFE2L2*, *CDKN2A*, *PIK3CA*, and *TP53* driver mutations.

## Materials and methods

### Ethics approval

Patients received both oral and written information and signed informed consent agreements as approved by the Regional Ethical Review Board in Gothenburg (Nos. #144‐13, #44‐18, #36‐2014, #1183‐2018, and #2023‐03923‐02). Anonymized key findings from diagnostic and clinical managements were collected from patient records.

Animal experiments were approved by the Regional Animal Ethics Committee in Gothenburg (Nos.: #36‐2014 and #1183‐2018) and performed in accordance with EU directive 2010/63.

### Patients and tumor sampling

Fresh tumor tissue samples for PDX studies were obtained after surgical resection of locally advanced thyroid cancers (*n* = 8) at Sahlgrenska University Hospital. Tissue sampling was done under sterile conditions immediately after surgery under supervision of a clinical pathologist. Tumor tissue pieces were cut into 1–2 mm^2^ fragments that were kept overnight in culture medium consisting of RPMI 1640 (Invitrogen; Thermo Fisher Scientific, Gothenburg, Sweden) and 10% heat inactivated Human AB serum (HS; Sigma Aldrich Solutions; Merck, Darmstadt, Germany) in 24‐well plates (Nunc Cell Culture; Thermo Fisher Scientific) before engraftment, as detailed below. Unless otherwise stated in the Results, clinical data of included patients are provided in supplementary material, Table S1.

### Genomic and mutation analyses

A target‐capture next‐generation sequencing (NGS) panel covering 560 genes (GMS560; https://genomicmedicine.se/wp‐content/uploads/2023/11/GMS560_synopsis.pdf) was employed for mutation profiling of tumors from patient 1 (Case 1). Whole‐exome sequencing (WES) was performed on DNA extracted from 1st passage (P1) xenograft biopsies of the primary tumor of patient 2 (Case 2) using the NucleoSpin Tissue kit (Macherey‐Nagel, Düren, Germany). Sequencing by Illumina (Illumina Inc, San Diego, CA, USA) using Agilent capture kits (Agilent Technologies, Santa Clara, CA, USA) was performed at the Genomics Core facility, University of Gothenburg. Variants were called using the GATK package (https://gatk.broadinstitute.org/hc/en-us/) (date last accessed 20/06/2024) [8] and annotated with Annovar (https://annovar.openbioinformatics.org/en/latest/) (date last accessed 21/03/2025) [9].

### Immunohistochemistry (IHC)

Formalin‐fixed paraffin‐embedded (FFPE) tissue sections collected on IHC Adhesion Microscope Slides (Tom‐11; Matsunami Glass Ind, Ltd, Osaka, Japan) were stained with hematoxylin‐eosin or immunolabeled with antibodies against (clone/number; dilution; source): CDH1/E‐cadherin (13‐1900; 1:4,000; Novex. Life Technologies; Thermo Fisher), CDH2/N‐cadherin (TA503933; 1:400; Thermo Fisher Scientific), Ki67 (ab15580; 1:100; Abcam, Cambridge, MA, USA), SNAI2 (ab27568; 1:200; Abcam), NRF2 (provided by Edward E. Schmidt lab; 1:100), NQO1 (HPA007308; 1:100; Sigma‐Aldrich), P40 (ACI3066C; 1:100; Biocare Medical, Pacheco, CA, USA) and P63 (DAT‐p63; FLEX/RTU; Dako; Agilent). Routine‐diagnostic IHC comprised: CK5/6 (D5/16 B4; Dako; Agilent), CK7 (OV‐TL 12/30; Dako; Agilent), CK19 (RCK108; Dako; Agilent), CD10 (mouse monoclonal clone 56C6, Dako; Agilent), and thyroglobulin (rabbit polyclonal IR509, Dako; Agilent). IHC was performed in a Dako Autostainer Link (Dako; Agilent) using the EnVision^TM^ FLEX detection system (HRP) following antigen retrieval in Dako PT‐Link using EnVision^TM^ FLEX Target Retrieval Solution (Dako; Agilent). Imaging used an Olympus BX45TF microscope (Olympus; Evident Scientific, Tokyo, Japan) equipped with a Nikon DS‐U2 digital camera (Nikon Corporation, Tokyo, Japan) and the NIS Elements Imaging software (https://www.microscope.healthcare.nikon.com/products/software/nis‐elements/) (date last accessed 25/08/2020).

### Patient‐derived xenografts and drug treatments

Small tumor pieces (*n* = 2/animal) were subcutaneously transplanted to 6–8 weeks old combined immune‐deficient interleukin‐2 chain receptor γ knockout mice (NOG mice; Taconic Biosciences, Lille Skensved, Denmark) housed in a pathogen‐free animal facility. Engrafted mice were kept in ventilated cages at room temperature with free access to sterilized food and water and a 7 am/pm dark–light cycle environment. PDX size was measured using a caliper every 2–3 days. P1 tumors were surgically removed from anesthetized mice and saved for analyses as detailed or retransplanted to generate P2 and P3 xenografts.

NOG mice bearing third‐passage (P3) PDXs were randomized into treatment groups (3–4 mice/group) and treated via oral gavage with small molecule inhibitors twice daily until sacrifice. Treatment groups included: vehicle (*n* = 3), cabozantinib (*n* = 4; 0.15 g/kg in standard mouse diet; Hospital Pharmacy, Sahlgrenska University Hospital), GDC‐0326 (*n* = 3; 12.5 mg/kg body weight; Sellek Chemicals, Houston, TX, USA), cabozantinib plus GDC‐0326 (*n* = 4), and CB‐839 (*n* = 4; 200 mg/kg body weight; HY‐12248; MedChemExpress, Monmouth Junction, NJ, USA). Vehicle contained 25% (w/v) hydroxypropyl‐b‐cyclodextrin in 10 mmol/l citrate buffer (pH 2.0). CB‐839 was formulated at 20 mg/ml for a final dosing volume of 10 ml/kg. Drugs were administered from the time xenografts became visible. PDX tumor growth response to drugs was evaluated by caliper measurements three times per week until sacrifice. PDX tumor biopsies (*n* = 2 per treatment group) were collected for IHC as indicated.

### Statistical analyses

One‐way analysis of variance (ANOVA) was applied for PDX endpoint size difference evaluation using GraphPad Prism v. 10 (https://www.graphpad.com/features), with data presented as mean ± SD and statistical significance set at *p* value less than 0.05.

## Results

### Synchronous squamous cell carcinomas confound tumor‐cell‐origin of thyroid SCC (Case 1)

**Figure 1:**
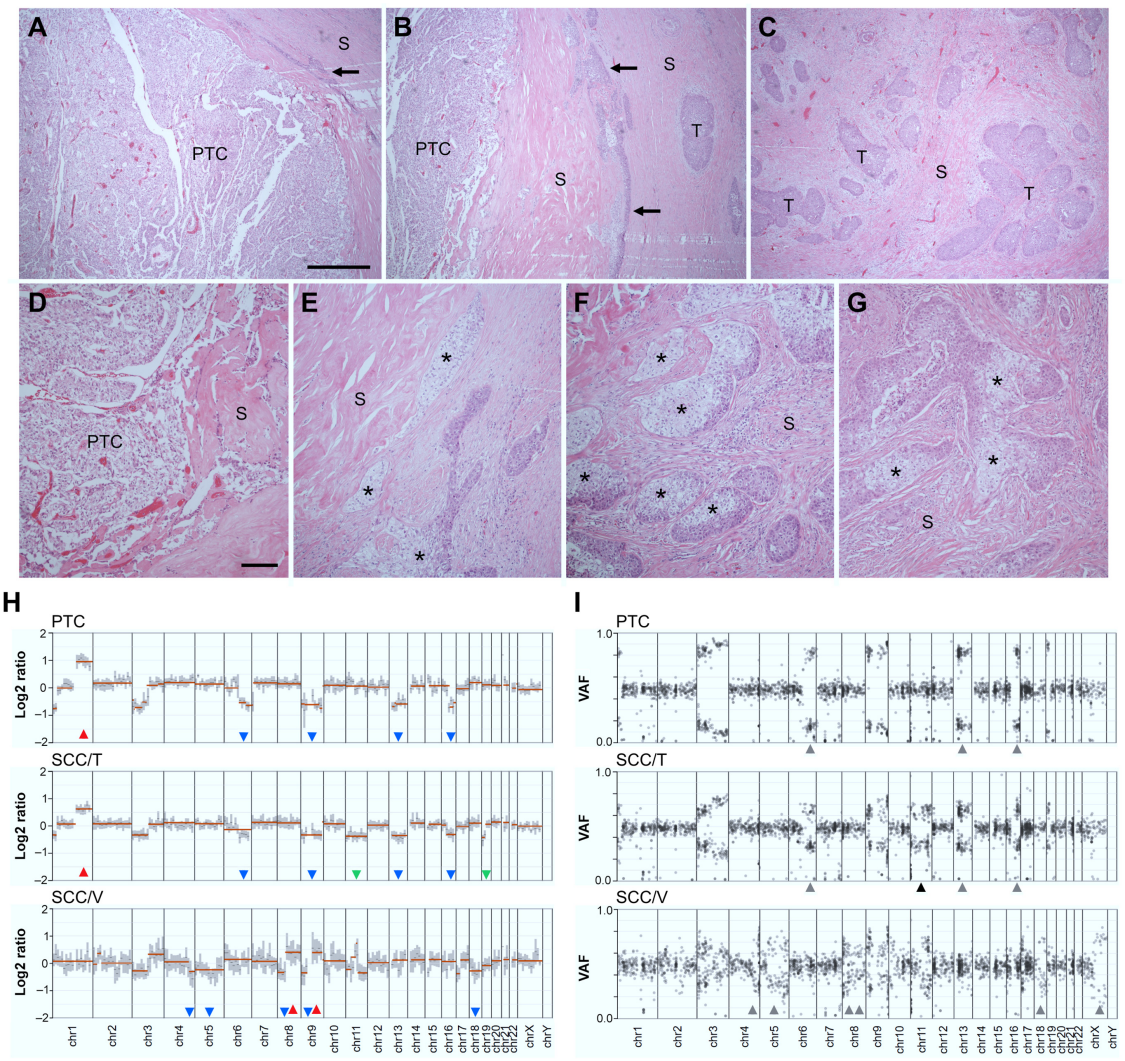
Squamous cell carcinoma (SCC) of the thyroid evolving from a papillary thyroid carcinoma (PTC). Routine hematoxylin–eosin staining of formalin‐fixed, paraffin‐embedded sections of a primary tumor present in the right thyroid lobe of a patient with concomitant vulvar cancer (Case 1). (A–C) Overview of (A) PTC, (B) PTC‐SCC interface, and (C) SCC tumor components embedded in abundance of fibrous stroma. (D–G) Tumor cell features in (D) PTC, (E, F) at PTC‐SCC interface zone and (G) SCC bulk tumor. Arrows indicate infiltrative squamous tumor portions. Asterisks indicate tumor cells with clear cytoplasm in squamous tumor lesions. T, tumor tissue; S, stromal tissue. Scale bars, 500 μm (panels A–C), 100 μm (panels D–G). (H, I) Concordant copy number alterations in PTC and SCC tumor components confirming a clonal relationship. Data from comprehensive targeted sequencing using the GMS560 panel on genomic DNA extracted from paraffin‐embedded sections of concurrent PTC and squamous cell carcinomas of the thyroid (SCC/T) and vulva (SCC/V) in the same patient. In (H): copy number profiles (log2 ratios); arrowheads indicate distinct copy number gains (red) and losses (blue or green). In (I): B‐allele frequencies (VAF); arrowheads indicate distinguishing features. In both: green and black arrowheads denote chromosome losses detected in SCC/T but not in the PTC tumor. Created by Affinity Designer 2 (affinity.serif.com).

In 2021, a 70‐year‐old female presented with a rapidly growing neck tumor (Case 1), classified as Bethesda category VI (malignant) by ultrasound‐directed fine‐needle aspiration cytology (FNAC). Following thyroidectomy with R2‐resection, the patient was diagnosed with a mixed PTC‐SCC (pT3N1aM0) in the right thyroid lobe (Figure 1A–C). Intriguingly, upon adjuvant treatment for thyroid cancer, comprising radioactive iodine (RAI) therapy (3.7 GBq) and external beam radiation (68 Gy) targeted to the thyroid bed, the patient was diagnosed with an advanced vulvar SCC with regional lymph node metastasis. This raised the possibility that the SCC thyroid tumor may in fact have originated from metastasizing gynecological cancer. After vulvar surgery, the patient received radiosensitizing chemotherapy with cisplatin and external beam radiation (50 Gy) directed to the inguinal lymph nodes. There were no clinical signs of recurrence in the thyroid. However, the vulvar SCC relapsed in July 2022, and the patient died in February 2023.

### Comprehensive targeted sequencing confirms transition of PTC to SCC

**Figure 2:**
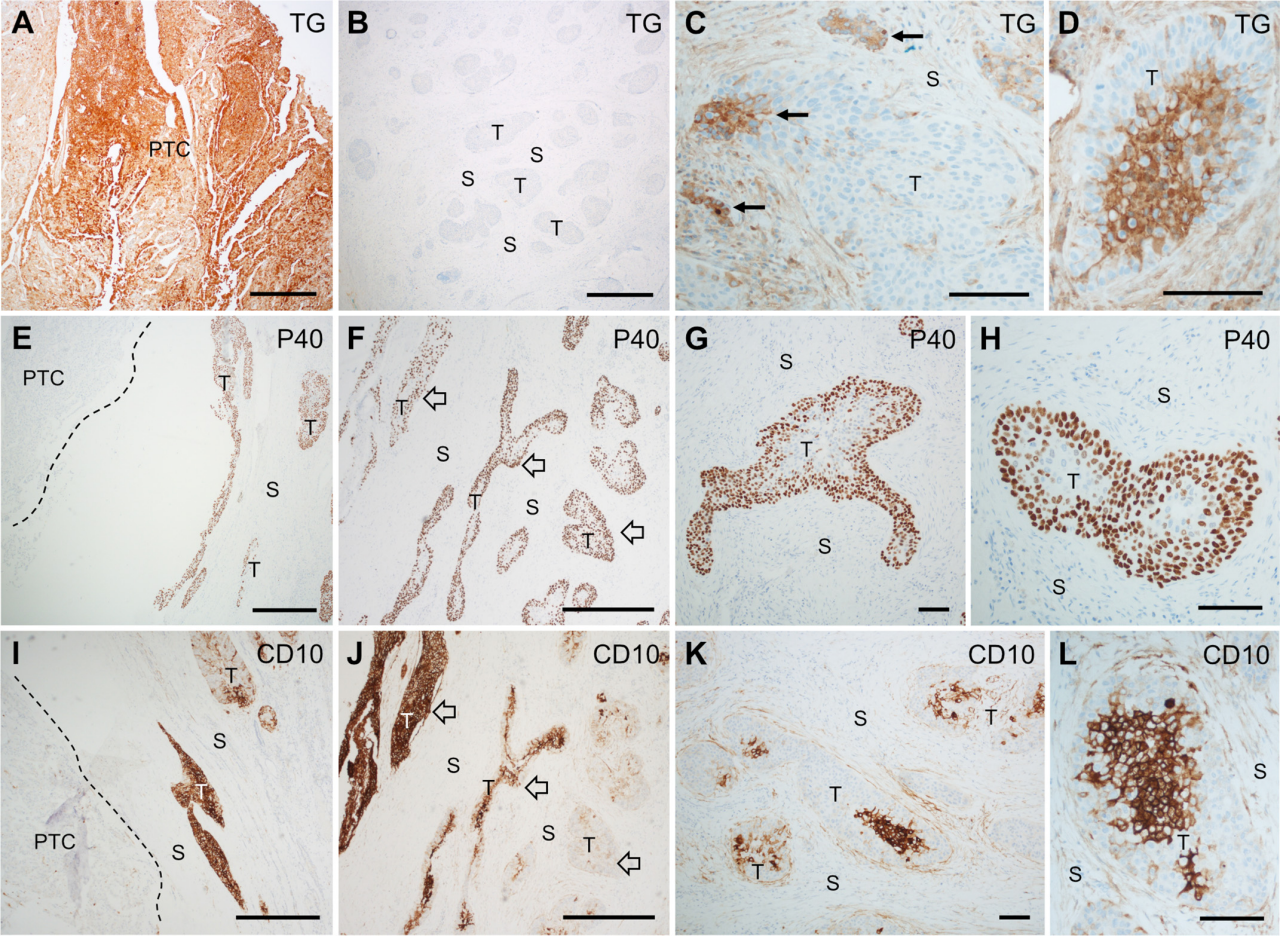
Tumor transition from papillary thyroid carcinoma (PTC) to squamous cell carcinoma (SCC). Immunohistochemical staining of thyroid tumor tissue sections of Case 1. (A–D) Differential expression of thyroglobulin (TG) in (A) PTC, (B) SCC bulk tumor, and (C, D) SCC tumor portions present in the vicinity of the papillary tumor. Arrows indicate TG^+^ cells. (E, F) P40 expression in clusters of SCC cells located (E) closest to the PTC and (F) slightly more peripheral to the papillary tumor, respectively. (G, H) Heterogeneous expression of P40 in SCC cell clusters. (I, J) Differential CD10 expression in SCC cells diminishing with increased distance from the PTC tumor. Dashed lines in panels (E) and (I) mark the PTC outer border. Open arrows in panels (F) and (J) indicate the same P40^+^/CD10^+^ motifs in serial sections for comparison. (K, L) Heterogeneous expression of CD10 within SCC clusters. T, tumor tissue; S, stromal tissue. Scale bars, 500 μm (panels A, B, E, F, I, J), 100 μm (panels C, D, G, H, K, L). Created by Affinity Designer 2 (affinity.serif.com).

The thyroid tumor of Case 1 measured 38 mm and contained a centrally located clear‐cell variant PTC, surrounded by multiple clusters of squamous tumor cells, presumably forming a coherent SCC network interspersed with an abundance of fibrous tissue (Figures 1A–H and 2E–H for SCC biomarker expression of P40). In addition to squamous cells, the SCC clusters included clear cells suggestive of a PTC‐SCC transition (Figure 1D–F). However, since both cell types were present adjacent to the PTC as well as in more peripherally located lesions, it is likely that this growth pattern reflects an established SCC tumor phenotype (Figure 1G).

To elucidate whether the thyroid and vulvar SCCs were clonally related or comprised synchronous primaries, a broad target‐capture NGS gene panel, developed within the framework of a national initiative for comprehensive mutation profiling of solid tumors (GMS560; https://genomicmedicine.se/wp-content/uploads/2023/11/GMS560_synopsis.pdf), was employed. Genomic DNA was obtained by careful microdissection of FFPE‐sections from both the vulvar and thyroid tumors. This revealed near‐identical copy number alternations in the histologically distinct thyroid tumors, whereas the gynecological tumor displayed different chromosomal aberrations (Figure 1H,I). Moreover, the two tumor components of the thyroid harbored identical mutations in *KEAP1*, *RB1*, and *STK11*, none of which were detected in the vulvar carcinoma (Table 1). The presence of *BAP1* and *PTPRC* mutations, along with the loss of one copy of chromosome 11 (Chr 11) predominantly or exclusively in the squamous tumor component, suggested that the thyroid SCC developed from the PTC, as further suggested by the gross tumor anatomy of SCC enclosing the PTC, rather than the reverse.

**Table 1:** Pathogenic and likely pathogenic mutations with variant allele frequency in coincidental carcinomas of the thyroid and vulva in a 70‐year‐old female patient (Case 1)

Mutated gene^a^	PTC	SCC/T	SCC/V
*KEAP1* p.L472del	82%	30%	‐
*STK11* p.K84*	80%	31%	‐
*RB1* c.1960+2T>C	64%	35%	‐
*BAP1* p.T310T^b^	0.9%	43%	‐
*PTPRC* p.Y444*	‐	7.9%	‐
*NOTCH1* p.R112fs*3	‐	‐	39%
*FAT1* pT1831Yfs*8	‐	‐	35%
*TERT* c.‐146C>T	‐	‐	30%
*RHOA* p.E40Q	‐	‐	30%
*HUWE1* c.4615‐10G>C	‐	‐	30%
*TP53* p.R175H	‐	‐	29%
*GTF2I* p.R866Vfs*8^c^	5.6%	3.9%	9%
*DNMT3A* p.K744Rfs*35^c^	5.6%	8.3%	7.3%

^a^ Based on sequencing using the GMS560 panel (https://genomicmedicine.se/wp‐content/uploads/2023/11/GMS560_synopsis.pdf) implemented at Sahlgrenska University Hospital.

^b^ Synonymous mutation predicted to encode a likely pathogenic splice variant.

^c^ *GTF2I* and *DNMT3A* mutations encountered at low frequency in all tumors likely represent clonal hematopoesis.

Abbreviations: PTC, papillary thyroid carcinoma; SCC/T, thyroid squamous cell carcinoma; SCC/V, vulvar squamous cell carcinoma.

### Biallelic inactivation of *KEAP1* , *STK11,* and *RB1* accompanies tumor progression from PTC to SCC

Sequencing data for chromosome 19p (Chr 19p), where the *KEAP1* gene is located, showed copy neutral loss of heterozygosity (CN‐LOH) in the PTC tumor component, associated with duplication of the mutant *KEAP1* allele and loss of the normal allele (Figure 1H,I). The SCC tumor component differed by an additional loss of one copy of Chr 19p. Notably, the *KEAP1* mutation locus (p.L472del) was located in a phylogenetically conserved KELCH domain involved in NRF2 binding [10], consistent with a likely loss‐of‐function effect. Therefore, in both thyroid tumor components, this may be potentially oncogenic due to the gain of NRF2 function. Indeed, recent pan‐cancer integrated genomic and functional studies demonstrate that *KEAP1* variants of unknown significance (VUS) can phenocopy established oncogenic *KEAP1* mutations [11].

Since the *STK11* gene is also located on Chr 19p, it is likely that the *STK11* K84 mutation also confers homozygous loss‐of‐function. Additionally, the loss of one copy of Chr 13, where *RB1* is located, was observed in both tumor components (Figure 1H,I). This LOH may provide conditions for the *RB1* splice site mutation, potentially contributing to PTC/SCC tumorigenesis.

Interestingly, both tumor components showed LOH of Chr 3p (Figure 1H,I), suggesting that a potential splice variant of mutant *BAP1*, predominantly found in the thyroid SCC tumor, may be pathogenic in the PTC‐SCC transition. Indeed, the *BAP1* p.T310T fraction (43%) was nearly identical to the expected number of cells carrying the mutation (based on the estimated fraction of 60% tumor cells in sampled tissue). The detection of 7/740 reads of the *BAP1* variant in the co‐occurring PTC (Table 1) further supports the notion that the squamous tumor in all probability originated from a PTC subclone.

Taken together, these findings clearly demonstrate that the coincidental thyroid and vulvar carcinomas are separate primary tumors, with the thyroid SCC developing from a preexisting PTC, which displayed an unusual mutation profile, comprising inactivation of multiple tumor suppressor genes. Notably, no *RAS* or *BRAF* mutation or *RET* fusions (analyzed by intron baiting of select genes, including *RET*) were encountered in the PTC tumor. From large TCGA datasets, it is evident that loss of Chr 11 is rare in PTC, but is frequent in head and neck and lung SCCs (supplementary material, Figure S1).

### Differential expression of thyroglobulin and CD10 identifies an intermediate PTC–SCC transition stage

The PTC tumor was generally positive for thyroglobulin (TG), whereas the main SCC component was TG‐negative (Figure 2A,B). However, some adjacent SCC clusters showed TG immunoreactivity, confined to a subpopulation of cells (Figure 2C,D), likely reflecting the differentiated state of the ancestral PTC. P40, the squamous‐specific isoform of P63 [12], was expressed throughout the entire SCC tumor, but not in the PTC (Figure 2E,F). However, P40 expression was not uniform. In the SCC clusters, the peripheral cells showed the strongest P40 immunoreactivity, while staining was almost absent in more centrally located cells (Figure 2G,H).

Both tumor components were positive for CK7 and CK19, whereas CK5/6 was weakly but uniformly expressed in SCC cells only (supplementary material, Figure S2A–C). Similarly, CD10, a membrane metallo‐endopeptidase implicated in tumorigenesis [16] and overexpressed in various malignancies, including thyroid carcinomas [13, 14, 15], was exclusively expressed in the SCC tumor (Figure 2I–L). However, CD10 was differentially expressed, with the highest levels in the SCC clusters located nearest to the PTC tumor, gradually diminishing with increasing distance from the PTC tumor (Figure 2I,J). Moreover, CD10 was predominantly expressed in the central portions of the SCC clusters (Figure 2K,L), in contrast to the expression pattern of P40. Heterogenous CD10 expression has been previously reported for tumors with varying levels of squamous differentiation [17, 18].

### Successful patient‐derived xenografting of a pure thyroid SCC (Case 2)

**Figure 3:**
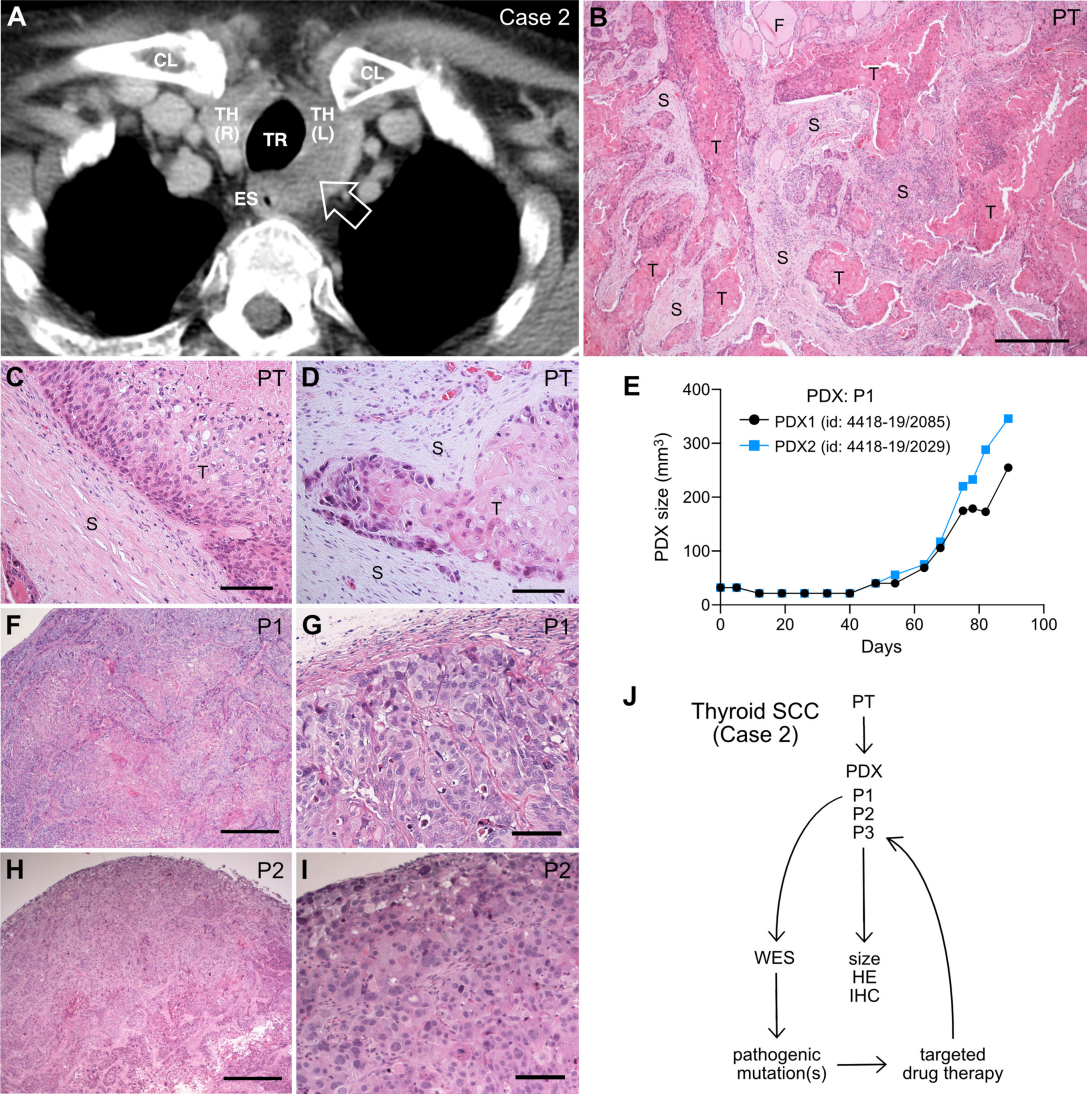
Radiologic imaging and histology of a pure thyroid squamous cell carcinoma (SCC) subjected to patient‐derived xenograft (PDX) experiments (Case 2). Hematoxylin–eosin (HE) staining. (A) Lower neck/upper thoracic CT scan. Open arrow indicates enlarged left thyroid lobe due to solid tumor growth extending dorsally towards the esophageal wall. (B–D) Primary tumor, overview of (A) tumor and stromal compartments (B), closeups of squamous tumor features (C), and invasive growth of tumor cells (D). (E) Growth kinetics of first passage (P1) PDX of the Case 2 SCC tumor subcutaneously engrafted onto NOG mice. Size changes were measured using calipers (*n* = 2; mouse ID numbers indicated). (F–I) Histological analysis of (F, G) P1 PDXs and (H, I) P2 PDXs, shown as low‐magnification overviews (panels F, H) and at high magnification (panels G, I). (J) PDX experimental setup. Workflow indicated by arrows comprised whole‐exome sequencing (WES) on P1, and drug treatment based on mutation profile on P3 transplants. TH(L), left thyroid lobe; TH(R), right thyroid lobe; TR, trachea; ES, esophagus; CL, clavicula; PT, primary tumor; T, tumor tissue; S, stromal tissue; F, follicles (adjacent to tumor); P1–P3, passage number of PDX. Scale bars, 500 μm (panels B, F, H), 100 μm (panels C, D, G, I). Created by Affinity Designer 2 (affinity.serif.com).

A second thyroid SCC (Case 2), diagnosed in 2019 in a 69‐year‐old male patient, was included in a limited PDX series, constituting the only primary tumor that was successfully engrafted out of eight advanced thyroid cancer specimens (supplementary material, Table S1). Preoperative computed tomography (CT) revealed a left thyroid lobe tumor that extended dorsally and was radiologically inseparable from the esophageal wall (Figure 3A). Tumor staging was pT3N1aM0. Neck surgery was not radical and adjuvant chemoradiotherapy (fractionated external beam radiation [68 Gy] and cisplatin/5‐FU) was administered without complications. The patient died in March 2020 due to metastatic disease progression.

Repeated endoscopy failed to confirm any tracheal, hypopharyngeal, or esophageal involvement, suggesting that the tumor‐cell‐origin was genuinely thyroidal, as confirmed by routine histopathological examination (Figure 3B). There were no signs of an adjoining DTC, consistent with the diagnosis of a pure thyroid SCC. The tumor cells showed clear signs of squamous differentiation and a tendency of keratinization in the central tumor portions (Figure 3C). Similar to the SCC in Case 1, the stromal component was extensive, with prominent signs of invasive tumor growth (Figure 3B,D).

**Figure 4:**
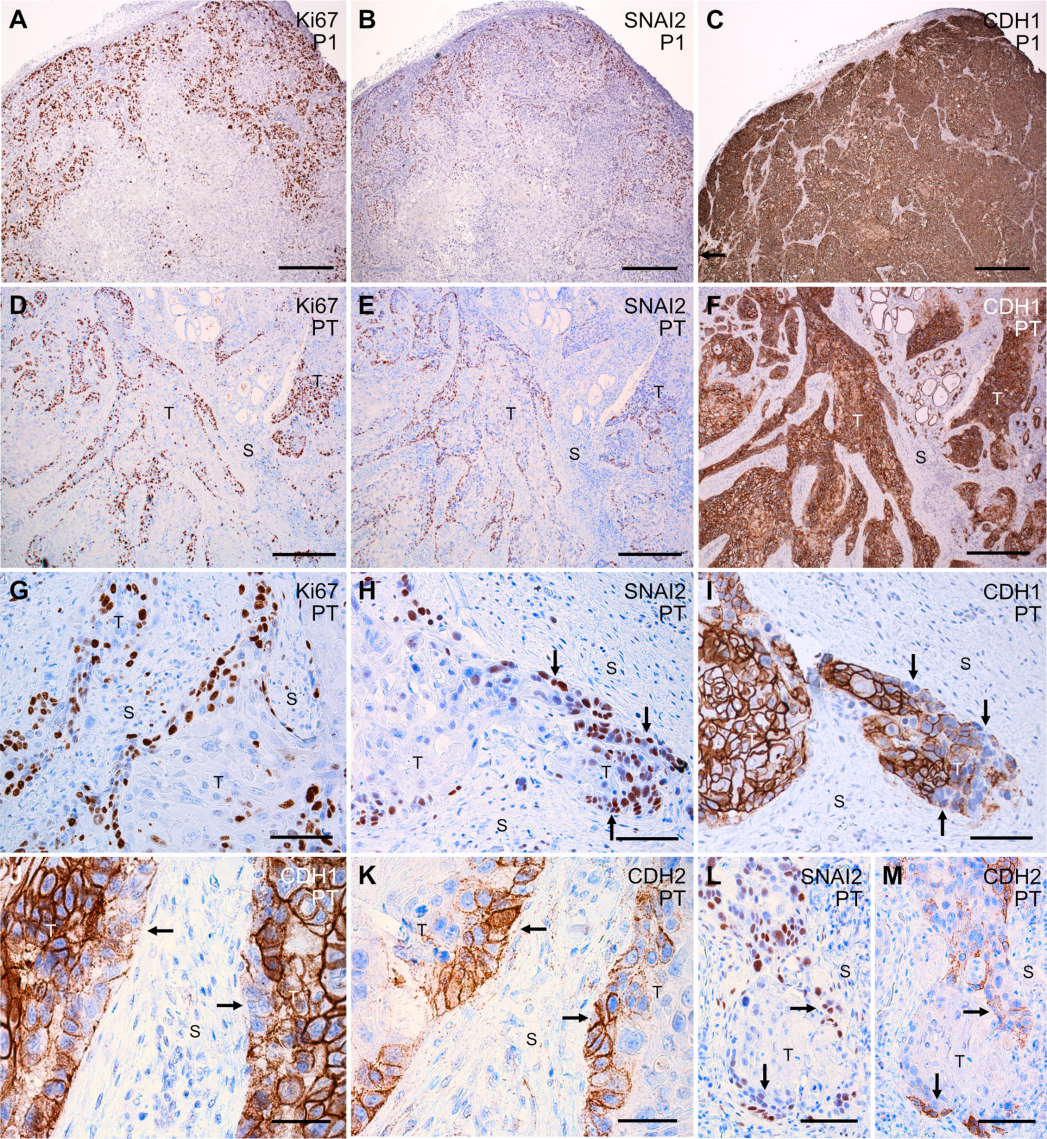
Biomarker expression of cell proliferation and epithelial‐mesenchymal transition (EMT) in PDX and primary tumor tissues of Case 2 thyroid squamous cell carcinoma (SCC). Immunohistochemical staining of: Ki67 (panels A, D, G), SNAI2 (panels B, E, H, L), CDH1/E‐cadherin (panels C, F, I, J) and CDH2/N‐cadherin (panels K, M). Serial sections allowing direct comparison are: (A–C), (D, E), (H, I), (J, K) and (L, M). (A–C) PDX, low‐magnification overviews. (D–M) Primary tumor, low‐magnification overviews (panels D–F), and high‐resolution images (panels G–M). Arrows indicate CDH1^low^/ SNAI2^+^/CDH2^+^ tumors cells facing the stromal compartment. For further details, see Results section. P1, first passage PDX; T, tumor tissue; S, stromal tissue. Scale bars, 500 μm (A–F), 100 μm (G–I, L, M), 50 μm (J, K). Created by Affinity Designer 2 (affinity.serif.com).

Eight primary tumor samples were engrafted into NOG mice (Table 1), adopting a protocol established for melanoma PDX [19]. Only the SCC transplants from Case 2 developed fast‐growing subcutaneous tumors, ~40 days after engraftment (Figure 3E). PDX tumor tissue was histologically more compact, with much less abundant stroma than in the primary tumor, and this growth pattern was consistent between passages (Figure 3F–I). Moreover, PDX cells showed little or no maturation towards a differentiated squamous phenotype. The differing growth pattern was further revealed by Ki67 expression, which was restricted to cells in the PDX periphery (Figure 4A), whereas in the primary thyroid tumor, Ki67^+^ cells were more widespread, although mainly located on the irregular tumor borders near the stromal tissue (Figure 4D,G). Primary and PDX tumors showed similar differences in the expression pattern of SNAI2 (Figure 4B,D,H), a member of the Snail family of transcription factors implicated in epithelial‐mesenchymal transition (EMT) [20]. However, it was only in the originating tumor tissue that SNAI2^+^ tumor cells displayed signs of EMT, shown by reduced E‐cadherin/CDH1 expression, which was largely restricted to cells at the tumor–stroma interface (Figure 4C,F,I). N‐cadherin/CDH2, a key EMT biomarker and effector [21], was enriched in primary tumor cells that showed upregulation of SNAI2 and diminished expression of E‐cadherin (Figure 4J–M).

Impaired restoration of the native tumor microenvironment presumably explained the more compacted PDX growth pattern, which was accompanied by a diminished stromal compartment and limited tumor invasiveness. We nonetheless decided to expand the mouse colony for targeted drug therapy on P3 xenografts based on driver mutation profiling of P1 tumor cells (Figure 3J).

### Targeted drug treatment inhibiting PDX growth of NRF2 mutant thyroid SCC cells

**Figure 5:**
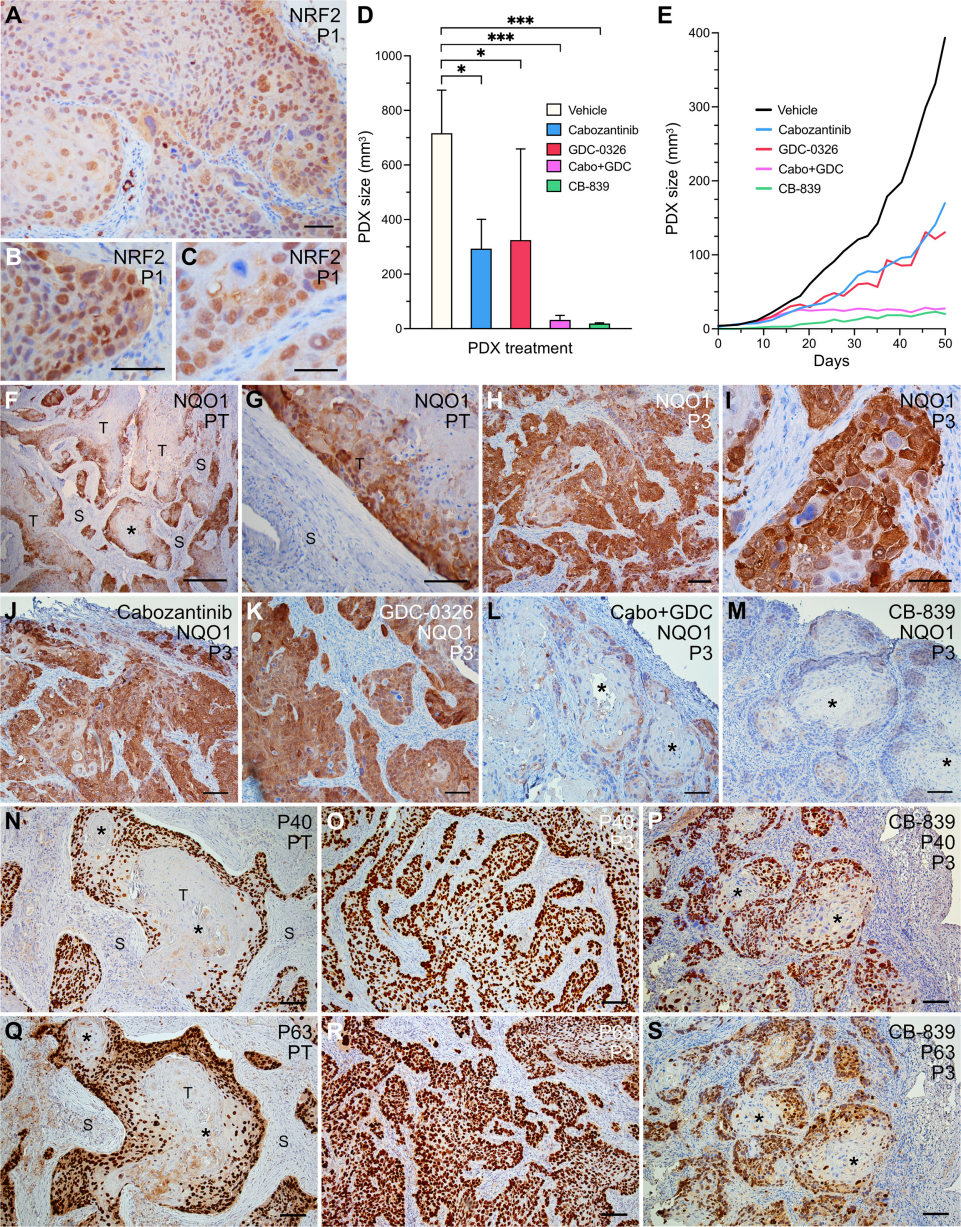
Nuclear localization of mutant NRF2 and targeted drug inhibition of growth acting downstream of *NRF2* activation in PDX derived from thyroid SCC (Case 2). Immunohistochemical staining of (A–C) NRF2, (F–M) NQO1, (N–P) P40 and (Q–S) P63; two retrieved grafts per treatment group were examined with similar results. Drugs administered orally on a daily basis for up to 50 days (onset when basal growth of engrafted tumors were detectable) were: cabozantinib (Cabo; 30 mg/kg), GDC‐0326 (GDC; 7.5 mg/kg), and CB‐839 (GLSi; 200 mg/kg). PDX size was monitored every 2–3 days. (A–C) NRF2 expression, low‐magnification overview (panel A) and high‐resolution images (panels B, C) of nuclear accumulation predominantly in tumor cells facing stromal tissue (i.e. the tumor edge). (D) Endpoint PDX size; mean ± SD (*n* = 3 in ‘vehicle’ and ‘GDC only’ groups; *n* = 4 in all other groups). *p < 0.05; ***p < 0.0002. (E) Kinetics of PDX enlargement; data from individual PDXs without (vehicle) or with drug treatment, as indicated. (F–I) NQO1 expression comparing (F, G) primary tumor and (H, I) PDX. (J–M) NQO1 expression changes in drug‐treated PDX. (N–S) Differential expression of P40 and P63 in (N, Q) primary tumor, (O, R) PDX without drug treatment and (P, S) PDX subjected to glutaminase inhibition. Asterisks (panels F, L, M, N, P, Q, S) indicate primary tumor/PDX areas with squamous differentiation designated by flattened cells and diminished NQO1 and P40/P63 expression. PT, primary tumor; P1, first passage PDX; P3, third passage PDX; T, tumor tissue; S, stromal tissue. Scale bars, 500 μm (panel F), 100 μm (panels A, B, G, H, J–S), 50 μm (panels C, I). Created by Affinity Designer 2 (affinity.serif.com).

PDX derived from the Case 2 thyroid SCC displayed pathogenic or likely pathogenic *CDKN2A*, *NFE2L2*, *PIK3CA*, and *TP53* mutations (Table 2). No concurrent *RAS* or *BRAF* mutations were found by WES. *NFE2L2* encodes NRF2, a KEAP1‐regulated transcription factor involved in oxidative metabolism and antioxidant defense [22, 23], commonly dysregulated in cancer cells [24, 25, 26]. Nuclear localization of mutant NRF2, likely reflective of constitutive NRF2 activation, was confirmed by IHC in the present PDX model (Figure 5A–C).

**Table 2:** Mutation profile of xenografted squamous cell carcinoma derived from the thyroid gland primary tumor in a 69‐year‐old male patient (Case 2)

Mutation^a^	Substitution	Significance
*CBLB* c.2200C>T	p.R734W	VUS
*CDKN2A* c.330G>A	p.W110*	Pathogenic
*FOXO1* c1373C>T	p.A458V	VUS
*NFE2L2* c242G>A	p.G81D	Likely pathogenic
*PIK3CA* c1633G>A	p.E545K	Pathogenic
*POLQ* c.7543+2T>A	possible splice	VUS
*TP53* c.824G>T	p.C275F	Pathogenic
*TSC2* c781C>T	p.R261W	VUS

^a^ Whole‐exome sequencing of a first‐passage (P1) patient‐derived xenograft (PDX).

Abbreviation: VUS, variant of uncertain significance.

Since increased NRF2 activity, resulting from *NFE2L2* or *KEAP1* mutations, sensitizes tumor cells to glutaminase inhibition, which reduces the bioavailability of glutamine otherwise serving as an antioxidant fuel for tumor growth and survival [6, 27, 28, 29, 30], mice bearing P3 xenografts were treated daily with CB‐839, a seective glutaminase inhibitor (GLSi) currently being evaluated in clinical trials with KEAP1/NRF2 mutant nonsmall cell lung cancer patients [31]. In parallel, PDX mice were similarly treated with multireceptor tyrosine kinase (RTK) inhibitor cabozantinib and PI3K inhibitor GDC‐0326, either alone or in combination. This resulted in a significant reduction in the endpoint PDX size for all drug treatments, although most efficiently with combined RTK and PI3K inhibition therapy, and GLSi monotherapy (Figure 5D). Retarded PDX growth was evident within 3 weeks for cabozantinib and GDC‐0326, and even earlier for CB‐839 (Figure 5E). Notably, the PDX size in these mice remained low until the end of the experiment, indicating sustained growth inhibition (Figure 5E).

### Diminished NQO1 expression accompanies drug‐induced SCC PDX growth inhibition consistent with impaired NRF2 signaling

NAD(P)H quinone oxidoreductase 1 (NQO1), an antioxidant enzyme involved in glutamine metabolism and a conserved NRF2 target gene [32], is overexpressed in many solid tumors, including SCC [33, 34, 35]. Since the NRF2 mutation is a likely driver event in Case 2, NQO1 expression was evaluated by IHC as a potential marker of NRF2 activity and predictor of response to targeted drug therapy. In the primary tumor, NQO expression was largely confined to the tumor cells facing the stroma (Figure 5F,G), similar to the distribution for Ki67 and SNAI2 expression (Figure 4D,E). In vehicle‐treated PDXs, NQO1 immunoreactivity was more evenly distributed among tumor cells (Figure 5H,I). Cabozantinib or GDC‐0326 alone did not influence NQO1 expression (Figure 5J,K). By contrast, both combined treatment with cabozantinib + GDC‐0326 and single‐drug treatment with CB‐839 reduced overall NQO1 expression and, notably, altered the distribution of NQO1 similar to the tissue distribution of NQO1‐positive and ‐negative cells in the primary tumor (Figure 5L,M; compare with Figure 5F). Changes in NQO1 expression therefore correlated with the level of PDX growth inhibition, irrespective of drug target, and likely reflected a general reduction in NRF2 activity in the growth‐inhibited tumor cells.

### Glutaminase inhibition promotes squamous differentiation in PDX‐grown thyroid SCC

Finally, we investigated whether efficient glutaminase inhibition downstream of NRF2 might influence the expression of SCC biomarkers, P40 and P63. In the primary tumor, both P40 and P63 showed a similar expression pattern as Ki67 and NQO1, and were essentially absent in the centrally located squamous portions (Figure 5N,Q). In untreated PDX, most, if not all, tumor cells were P40/P63‐positive (Figure 5O,R). CB‐893 treatment reproduced a heterogeneous expression of P40 and P63, typical of squamous differentiation (Figure 5P,S), closely resembling the NQO1 expression pattern (Figure 5M). Therefore, drug‐induced differentiation of thyroid SCC cells is likely a result of diminished NRF2 activity.

## Discussion

**Figure 6:**
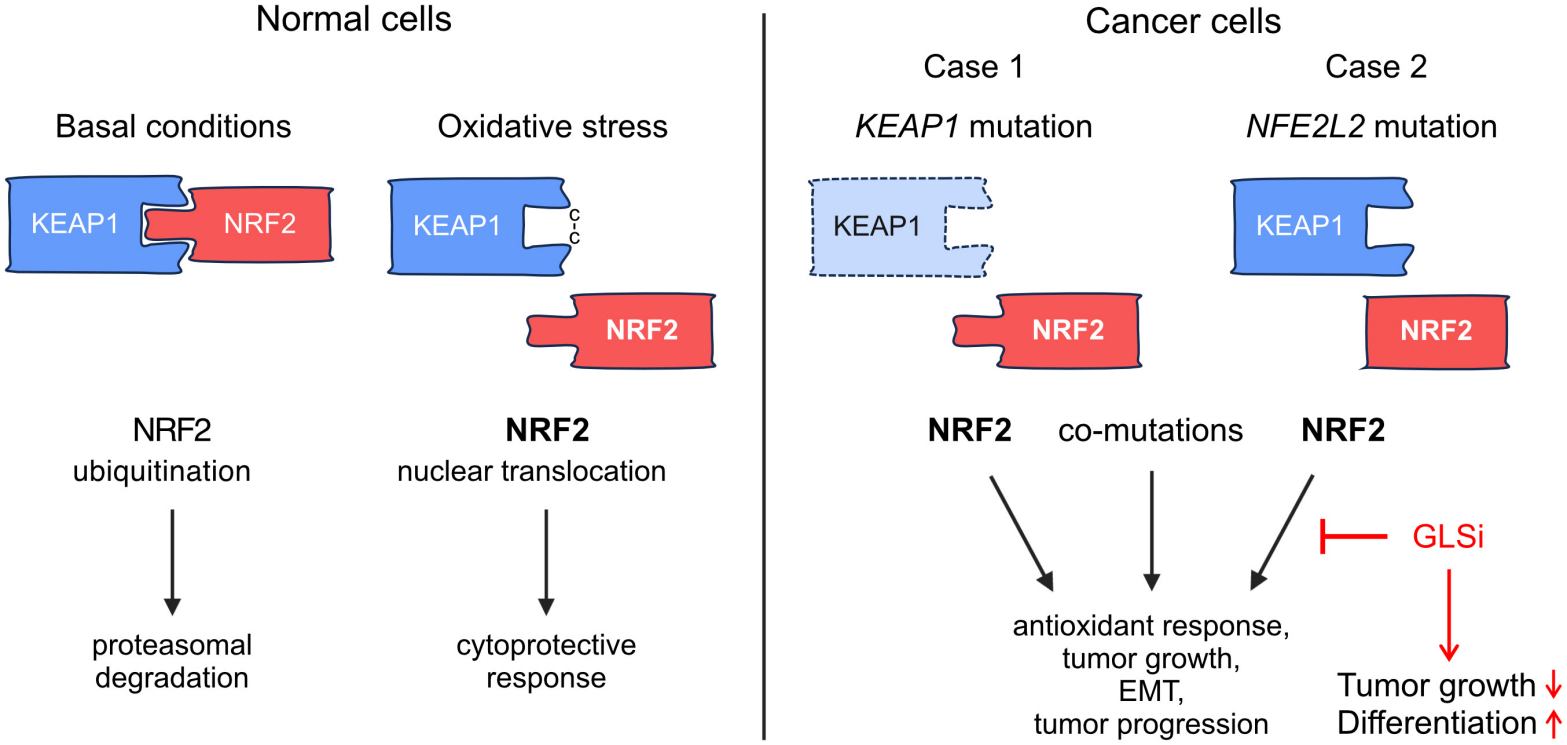
Summary of present findings suggesting a role of *KEAP1* and *NFE2L2* mutations in the pathogenesis of squamous cell carcinoma (SCC) of the thyroid, a subtype of anaplastic thyroid carcinoma (ATC), and the potential therapeutic value of inhibiting metabolic rewiring due to increased NRF2 activity in SCC tumor cells. Left panel: In normal cell homeostasis, KEAP1 facilitates proteasome‐dependent degradation of NRF2, thereby silencing NRF2 transcriptional activity. In response to oxidative stress, this function of KEAP1 is inhibited, leading to the accumulation of NRF2, which enters the nucleus and elicits a cytoprotective transcriptional program [23]. Right panel: In NRF2‐addicted cancer cells, NRF2‐mediated transcription is derepressed due to *KEAP1* or *NFE2L2* mutations, which prevent NRF2 from entering the natural degradation pathway. The resulting constitutive NRF2 activation drives metabolic reprogramming of tumor cells, associated with loss of growth control and a malignant state [91], which may be counteracted by drug targeting of the involved signaling pathways [92]. Case 1 and Case 2 represent suggested scenarios based on patient and PDX data obtained in the present study, which showed that a glutaminase inhibitor (GLSi) exerts a profound growth‐inhibitory effect along with enhanced squamous differentiation in NRF2 mutant thyroid SCC tumor cells. Conceivably, GSLi or other approaches to block NRF2 activity should be considered as potential adjuvant treatment strategies for patients with thyroid SCC lacking other therapeutic options. Created by Affinity Designer 2 (affinity.serif.com).

This study provides the first direct evidence of tumor progression from PTC to SCC, likely created by sequential loss‐of‐function of tumor suppressor genes (*KEAP1* and *STK11*), in humans. We also provide proof‐of‐principle that NRF2‐induced metabolic rewiring plays a central role in proliferation and differentiation of thyroid SCC cells. Targeting metabolic vulnerabilities downstream of NRF2 activation by glutaminase inhibition offers dual benefits, by inhibiting tumor growth and promoting squamous differentiation, which is consistent with a less invasive phenotype (Figure 6).

As recently demonstrated by whole‐genome sequencing [36], co‐occurring ATC and DTC often share a clonal origin, confirming the generally accepted view that ATC may progress from PTC or a follicular thyroid carcinoma (FTC). The origin of thyroid SCC, classified as an ATC variant since 2022 [1], has been challenging to determine unless a *BRAF* mutation is present, suggesting derivation from PTC [3]. Notably, there are several possible tumor‐cell‐origins of SCC other than the thyroid follicular cell [37], including the ultimobranchial bodies, which consist of a stratified epithelium before merging with the thyroid proper [38, 39]; ultimobranchial‐derived follicles and solid cell nests [40, 41, 42, 43, 44, 45, 46, 47, 48]; and thyroglossal duct remnants in which PTC and SCC may coexist [49, 50, 51, 52, 53]. Thyroglossal duct‐derived cysts can be found ectopically within the thyroid gland [54]. Any of these sources could potentially serve as the origin of SCC, especially in the absence of a concurrent DTC.

We demonstrated the transition of PTC to SCC, in which tumor cells carried none of the common driver mutations (e.g. *BRAF* or *RAS*), but showed biallelic inactivation of *KEAP1*, *STK11*, and *RB1*. KEAP1 inactivation is a known contributor to lung cancer [6], and KEAP1 zygosity was recently found to predict drug sensitivity and therapeutic response [11]. *KEAP1* mutations have been previously reported to occur in a small subset of PTC [55]. However, the incidence of *KEAP1* mutations is generally lower in PTC and ATC in comparison to nonthyroid SCC (TCGA datasets; cBioPortal), although aberrations in *KEAP1* and *CUL3*, another key component of proteasome‐mediated NRF2 degradation [56], specifically accumulate in ATC tumors (supplementary material, Table S2). Abnormal activation of the NRF2 pathway is nonetheless frequent in PTC and may involve dysregulation of the NRF2 inhibitor complex due to epigenetic modifications [57, 58]. Experimental models have that Keap1 and P53 co‐inactivation elicits SCC development in airway basal stem cells, but not in respiratory epithelial cells [7], suggesting that the acquired tumor phenotype depends on the differentiation state of the ancestor mutant cell. Similarly, gain‐of‐function *Nfe2l2* mutation, targeted to the esophagus, triggers SCC tumorigenesis, whereas *Keap1* loss‐of‐function with mutant *Trp53* does not [59]. Therefore, KEAP1 inactivation alone cannot confer tumor development or progression towards the SCC thyroid phenotype.

*STK11*, encoding serine/threonine kinase 11, is an established tumor suppressor gene and inactivating mutations occur in many different tumors. Germline *STK11* mutations cause Peutz–Jeghers syndrome (PJS) and confer increased thyroid cancer risk [60]. Mutated *STK11* occurs in 10% of pediatric DTC [61], and may occur in PTC and FTC in adults without PJS [62]. Notably, loss of Stk11/Lkb1 promotes SCC development in mouse lung cancer models [63, 64, 65, 66, 67, 68]. *KEAP1* and *STK11* co‐mutation may therefore potentially predispose to progression of PTC to SCC. *RB1* inactivation has also been implicated in squamous tumor development [69, 70, 71].

Solid tumors with alterations in BAP1 are often associated with squamous histopathology [72]. Moreover, in lung cancer *BAP1* is tumor‐suppressive by inhibiting the KEAP1/NRF2 signaling pathway [73]. In Case 1 of the present study, tumor cells progressing to SCC acquired *BAP1* mutation close to a splice‐donor/acceptor consensus motif, which in the concurrent absence of a wildtype *BAP1* allele (due to Chr 3p LOH), would be sufficient to accomplish homozygous loss‐of‐function and potentially contribute to subclonal tumor progression. The significant rarity of thyroid SCC and a surprisingly high prevalence of Chr 3p LOH in DTC (40% of PTC and 67% of FTC) [74] nonetheless predicts that pathogenic *BAP1* mutations must be rare events in thyroid malignancy.

*NFE2L2* mutations are prevalent in SCC of the skin, esophagus, and lung [7, 75]. However, public sequencing data indicate that *NFE2L2* mutations do not occur in PTC, although some ATCs harbor *NFE2L2* mutations (2.6%) and amplification (6.3%) (supplementary material, Table S2), which together confirm the rarity of thyroid SCC. However, since NRF2‐mediated antioxidant responses confer a viability advantage to PTC tumor cells [76, 77], it is plausible that abnormal NRF2 activation may be more frequent, and that targeting the *KEAP1*/*NRF2* pathway (e.g. by glutaminase inhibition) may be beneficial to patients with advanced thyroid cancer other than SCC. Our findings further support that NQO1 IHC is a robust marker of NRF2 activation [78], and may potentially be used for patient screening of amplified NRF2 signaling and potential responsiveness of residual or relapsing tumors to GLSi.

High NQO1 expression, localized to SNAI2^+^/CDH1^low^/CDH2^high^ cells at the invasive front, facing the tumor stroma, is consistent with recent findings that NQO1 supports a sustained EMT response by stabilizing Snail transcriptional activity [79, 80, 81]. Conversely, NQO1 downregulation in cells undergoing a switch to a more differentiated squamous phenotype following drug treatment, resembles natural squamous differentiation, which is characterized by reduced expression of P40 and P63 [82, 83]. This further supports the rationale for targeting downstream effects of mutant *NRF2* by glutaminase inhibition. Similar results achieved by TRK and PI3K inhibitors are unsurprising, as multiple signaling pathways may alter tumor cell metabolism and NRF2‐mediated NQO1 activation through different mechanisms [79, 81, 84].

It is remarkable that out of eight advanced thyroid malignancies subjected to xenotransplantation only one tumor, a SCC, was successfully propagated. For comparison, the PDX success rate is nearly 90% for melanomas using a similar protocol [19]. A low xenograft take of ATC is in line with previous efforts [85]. This underscores that thyroid SCC differs significantly from other ATC tumors and suggests that subtyping based on comprehensive mutation analysis is likely of clinical value, serving as guidance for adjuvant targeted drug treatment. In view of the present findings of the beneficial effects of pharmacological inhibition, downstream of NRF2 signaling, in thyroid SCC tumor cells in a PDX model, and accumulating evidence of NRF2 as a central player and mediator of chemoresistance [86, 87, 88], we suggest that glutaminase inhibitor, or other drugs being developed to interfere with the KEAP1/NRF2 pathway, should be considered in clinical trials to improve the treatment of patients with advanced thyroid cancer that have an otherwise poor prognosis.

## Author contributions statement

MN, JAN and VIS were responsible for the conceptualization of the study. ES, ND, EA, LMN, JAN and MN designed and carried out experimental work. ND and EA developed softwards andES, LMN, GM and HF undertook validation studies. ES, JD, JJDz, ND, EA, LMN, TC and GM were responsible for further data investigation. Resources to support the study were provided by GM, AM, JAN, VS and MN. Data Curation was undertaken by JD, ES, ND, EA, HF, JAN and MN. The original manuscript draft was prepared by MN, with review and editing by ES, JD, HF, AM, EE, VIS, JAN and MN. Figures were produced by ES, JJD, LMN, EE, JAN, MN and HF with overall supervision by MN, AM, EE and JAN. Administrative support was provided by MN and JAN. Funding was secured by MN, JAN, VIS, ES and JD. All authors reviewed and agreed to the final manuscript.

## Supporting information


**Figure S1.** Copy number variations in squamous cell carcinomas of nonthyroid origin and in papillary thyroid carcinomas
**Figure S2.** Cytokeratin expression in a compound squamous cell and papillary thyroid carcinoma
**Table S1.** Clinical data of patients with advanced thyroid cancer subjected to patient‐derived xenografting
**Table S2.** Occurrence of mutations involving the KEAP1/NRF2 pathway in anaplastic thyroid carcinoma in comparison to squamous cell carcinomas of other organs

## Data Availability

Experimental data that support the finding of this study are available from the corresponding author (MN) upon request. Public data supporting the findings are available at: https://www.cbioportal.org/
