# Supplementary material for: Involvement of KEAP1/NRF2 pathway in non‐BRAF mutated squamous cell carcinoma of the thyroid
Source: J Pathol. 2025 Jul 2;266(4-5):481–94. doi: 10.1002/path.6444 (PMC12256384; doi:10.1002/path.6444)
Supplement: Supplementary file 1 — Figure S1. Copy number variations in squamous cell carcinomas of nonthyroid origin and in papillary thyroid carcinomas Figure S2. Cytokeratin expression in a compound squamous cell and papillary thyroid carcinoma Table S1. Clinical data of patients with advanced thyroid cancer subjected to patient‐derived xenografting Table S2. Occurrence of mutations involving the KEAP1/NRF2 pathway in anaplastic thyroid carcinoma in comparison to squamous cell carcinomas of other organs [file PATH-266-481-s001.docx]

**Involvement of KEAP1/NRF2 pathway in non-BRAF mutated squamous cell carcinoma of the thyroid**

E Schoultz *et al.* *J Pathol* <https://doi.org/10.1002/path.6444>

**Supplementary Figures S1 and S2**

**Supplementary Tables S1 and S2**

**Reference numbers refer to the main text list**

**Figure S1. Copy number variations in squamous cell carcinomas of nonthyroid origin and in papillary thyroid carcinomas.** Data were obtained from the following TCGA Firehose Legacy datasets via cBioPortal: Head and Neck Squamous Cell Carcinoma (<https://www.cbioportal.org/study/summary?id=hnsc_tcga>), Lung Squamous Cell Carcinoma (<https://www.cbioportal.org/study/summary?id=lusc_tcga>), and Papillary Thyroid Carcinoma (<https://www.cbioportal.org/study/summary?id=thca_tcga>. Blue indicates copy number loss and red indicates copy number gains. Copy number profiles of the thyroid tumor with squamous differentiation (SCC/T) of Case 1 are shown in the top panel (identical to Figure 1H) for reference. Chromosomes 11 and 19, which show copy number loss in the SCC/T, but not in the corresponding PTC component, are marked by rectangles in the middle and bottom panels. HNSCC, head and neck squamous cell carcinoma (corresponding to TCGA term HNSC); LSCC, lung squamous cell carcinoma (corresponding to TCGA term LUSC); PTC, papillary thyroid carcinoma.

**Figure S2. Cytokeratin expression in a compound squamous cell and papillary thyroid carcinoma.** Immunohistochemical staining of (A) cytokeratin (CK)-19, (B) CK7, and (C) CK5/6 in serial sections of primary tumor referring to Case 2, for which mutation profiling revealed progression of squamous cell carcinoma (SCC) from differentiated thyroid cancer. The border between the main tumor components is outlined. Arrows indicate the same SCC tumor lesion being recovered in all three images. Scale bar, 500 µm (applies to all images). PTC, papillary thyroid carcinoma; T, tumor tissue (squamous); S, stromal tissue.

**Table S1.** Clinical data of patients with advanced thyroid cancer subjected to patient-derived xenografting

| **Code/age/sex^1^** | **Diagnosis^2^** | **Stage^3^** | **Adjuvant treatment^4^** | **Outcome^5^** | **PDX^6^** |
| --- | --- | --- | --- | --- | --- |
| 441813/89/F | PDTC | T4N1bM1 | RAI 7.4 GBq | DOD | No take |
| 441814/47/F | ATC* | T4N1bM0 | EBRT 45 Gy + Paclitaxel | DOD | No take |
| 441815/70/F | FTC | T3NxM0 | RAI 3.7 GBq x2 + reop. | AWD | No take |
| 441819/69/M | SCC*** | T3N1bM0 | EBRT 68 Gy + cisplatin/5-FU | DOD | +after 40 days |
| 441822/85/F | PTC | T1bN1bM0 | RAI 3.7 GBq | NED | No take |
| 441823/35/M | PTC | T3N1bM0 | RAI 3.7 GBq | NED | No take |
| 441822/40/F | PTC | T2(m)N1bM0 | RAI 3.7 GBq | NED | No take |
| 44186/69/F | MTC** | T1bNxM1 | Temozolomide + capecitabine | DOD | No take |

^1^F, female; M, male.

^2^Based on histopathology/IHC; PTC, papillary thyroid carcinoma; FTC, follicular thyroid carcinoma; MTC, medullary thyroid carcinoma; PDTC, poorly differentiated thyroid carcinoma; ATC, anaplastic thyroid carcinoma; SCC, squamous cell carcinoma.

^3^According to the 8^th^ edition of the TNM staging system of the American Joint Committee on Cancer (AJCC) [89].

^4^RAI, radioiodine; EBRT, external beam radiation therapy; FU fluorouracil

^5^DOD, dead of disease; AWD, alive with disease; NED, no evidence of (residual/recurrent) disease; ^6^Observation time, 12 months for all “No take” transplants.

*PTC-derived; ***RET* mutation negative + calcitonin negative; ***Case 2 in present study.

**Table S2.** Occurrence of mutations involving the KEAP1/NRF2 pathway in anaplastic thyroid carcinoma in comparison to squamous cell carcinomas of other organs

| **Gene** | **PTC^1^**  **(*n*=399)** | **ATC^2^**  **(*n*=190)** | **PanCancer^3^**  **(*n*=2,658)** | **HNSCC^1^**  **(*n*=528)** | **LSCC^1^**  **(*n*=511)** | **ESCC^1^**  **(*n*=186)** |
| --- | --- | --- | --- | --- | --- | --- |
| ***KEAP1*** |  |  |  |  |  |  |
| Mutation | 0.2% | 0.5% | 1.2% | 4.3% | 12.4% | 3.2% |
| Homdel | 0.0% | 4.2% | 0.6% | - | 0.2% | 0.5% |
| Amp | 0.0% | 11.1% | 4.5% | 1.0% | 1.4% | 1.6% |
| ***CUL3*** |  |  |  |  |  |  |
| Mutation | 0.2% | 0.5% | 0.8% | 3.1% | 6.2% | 1.6% |
| Homdel | 0.2% | 11.1% | 1.1% | 2.5% | 1.8% | 1.1% |
| Amp | 0.0% | 5.3% | 0.2% | - | - | 0.5% |
| ***NFE2L2*** |  |  |  |  |  |  |
| Mutation | 0.0% | 2.6% | 1.7% | 5.3% | 15.2% | 8.6% |
| Homdel | 0.2% | 9.5% | 0.1% | - | 0.6% | 0.5% |
| Amp | 0.0% | 6.3% | 2.2% | 4.8% | 5.0% | 4.3% |

Homdel, Homozygous deletion; Amp, Amplification; PTC, Papillary thyroid carcinoma; ATC, Anaplastic thyroid carcinoma; PanCan, Pan-cancer; HNSCC, Head/neck squamous cell carcinoma; LSCC, Lung squamous cell carcinoma; ESCC, Esophageal squamous cell carcinoma.

^1^TCGA Firehose Legacy data (<https://www.cbioportal.org/>).

^2^GATCI data [36].

^3^ICGC data [90].
